# Evidence Drift in Early Childhood Caries Research: A Conceptual Six-Domain Causal-Translation Framework

**DOI:** 10.3390/children13081053

**Published:** 2026-08-07

**Authors:** Ziad D. Baghdadi

**Affiliations:** 1 TopSmiles Pediatric Dentistry & Orthodontics, 246 St. Anne’s Road, Winnipeg, MB R2M 3A4, Canada; drziadeddin.albaghdadi@gmail.com; 2 Butterfly Dental Group, Winnipeg, MB R3Y 1P5, Canada; 3 Children’s Dental Centre, 1630 Ness Ave, Winnipeg, MB R3J 3X1, Canada

**Keywords:** early childhood caries, evidence drift, causal inference, conceptual framework, narrative review, evidence appraisal, implementation science, health equity, silver diamine fluoride, commercial determinants of health

## Abstract

**Highlights:**

**What are the main findings?**

• Evidence drift occurs when findings are used to support claims across evidentiary domains (association, mechanism, causation, consequence, disease control, policy implementation) without adequate bridging evidence.
• Five case studies (vitamin D, GA rehabilitation, microbiome, SDF, and sugar taxation) illustrate distinct drift trajectories and competing interpretations in early childhood caries literature.

**What is the implication of the main finding?**

• The conceptual framework provides a transparent vocabulary, a four-question decision procedure, and testable hypotheses to improve inter-rater reliability during peer review, guideline development, and evidence appraisal.

**Abstract:**

Background/Objectives: Early childhood caries (ECC) is a common, preventable, and socially patterned disease, yet the literature on ECC is vulnerable not only to limitations in evidence generation but also to errors in evidence translation. This conceptual framework paper focuses on evidence translation rather than estimating a new treatment effect. Methods: Literature and framework development were informed by targeted narrative searches of PubMed/MEDLINE, Scopus, Web of Science, and Google Scholar, with an emphasis on ECC literature from 2010 onward and foundational methodological sources. Sources were purposively retained if they clarified an evidentiary domain, a cross-domain bridge, or a competing interpretation; priority was given to systematic reviews, trials, longitudinal and causal studies, natural experiments, clinical guidance, and implementation evaluations. No pooled effect estimates were produced. Results: Evidence drift is the movement of a finding into a stronger or different claim without adequate bridging evidence. The proposed framework comprises association, mechanism, causation, consequence, disease control, and policy implementation, with commercial and structural determinants operating as a cross-cutting upstream layer. It classifies the inference advanced rather than study design alone and permits primary and secondary domain tags. A four-question test asks whether the research question and conclusion occupy the same domain, what bridge supports the movement, and whether uncertainty is retained. Five trajectories illustrate the framework: vitamin D, dental rehabilitation under general anesthesia, oral microbiome research, silver diamine fluoride (SDF), and sugar taxation. In the SDF case, lesion-arrest evidence is explicitly separated from the still-unproven implementation hypothesis that endpoint-only pathways may create differential standards of care. Conclusions: The original contribution is an integrated vocabulary, decision procedure, and validation agenda for ECC evidence appraisal. The framework generates three prespecified, testable hypotheses: (1) trained raters will classify claims with reproducible inter-rater agreement (kappa ≥ 0.70); (2) claims judged to contain evidence drift will be significantly less likely than domain-concordant claims to include an explicit bridge; and (3) using the structured framework will improve the transparency and proportionality of reviews compared with usual appraisal. Prospective testing of content and construct validity, as well as practical utility, in peer review, guideline development, education, and policy evaluation is required before standardized implementation.

## 1. Introduction

Early childhood caries (ECC) is a biofilm-mediated, sugar-driven, multifactorial disease shaped by feeding patterns, free-sugar exposure, fluoride availability, oral hygiene, enamel and salivary factors, microbial ecology, caregiver capacity, poverty, access to dental care, and broader social and structural determinants [1,2,3,4]. Because ECC is clinically visible in teeth yet socially patterned within families and communities, research in this field must be especially careful when moving from observation to explanation and from intervention effects to policy recommendations.

The ECC scholarship has expanded into nutrition, microbiology, treatment outcomes, minimally invasive care, implementation science, and public health policy. The interpretive risk is that results answering one question are used to answer another: vitamin D associations [5,6] may be extended to prevention claims despite causal findings [7,8,9]; cross-sectional microbiome profiles may be interpreted as causal initiation [10,11,12,13]; or lesion-arrest outcomes may be treated as completion of care [14,15]. These are not defects in the underlying studies when their claims remain calibrated; the concern arises when later interpretation exceeds what the design and outcome can establish.

This interpretive slippage is termed evidence drift. Evidence drift occurs when findings from one evidentiary domain are used, implicitly or explicitly, to support conclusions in another domain without an adequate bridge. It is distinct from scientific disagreement or the normal revision of knowledge as evidence accumulates; the problem is not translation itself, but translation that obscures what a design, outcome, or intervention can reasonably demonstrate.

Evidence drift matters because domain confusion can alter research priorities, guidelines, and care pathways. For children, a scalable disease-control intervention may be valuable even when diagnosis, prevention, monitoring, referral, and definitive treatment are still required when indicated. The framework therefore separates the demonstrated effect from the additional evidence required for broader causal, clinical, or policy claims [1,2,16].

This Communication is a conceptual framework paper, not an original clinical study or a systematic review. Its objectives are to (1) define evidence drift, (2) develop a six-domain causal-translation framework, (3) demonstrate its application through five purposively selected ECC trajectories, and (4) specify testable procedures for validation and implementation. Its contribution is methodological—a common language and appraisal process—rather than new estimates of ECC risk or treatment effect.

## 2. Conceptual Approach, Literature Selection, and Scope

This paper was developed as a theory-building synthesis informed by a targeted narrative review. The unit of analysis is the inference linking a research question, design, outcome, conclusion, and recommendation; the framework does not classify study design in isolation. Because the purpose is conceptual development rather than effect estimation, the review was not designed as a systematic review or meta-analysis and does not claim exhaustive coverage [17,18,19].

### 2.1. Development Questions and Original Contribution

Three development questions guided the synthesis: (1) Can ECC evidence be classified by the primary inferential or clinical question it addresses? (2) What bridging evidence is required when a conclusion moves across domains? (3) Can a brief appraisal procedure make such movement visible to researchers and decision-makers? The original contribution is the integration of six domains, a primary/secondary tagging rule, the Four-Question Evidence Drift Test, a cross-cutting upstream-determinants layer, and an explicit validation agenda.

The framework generates three prospective hypotheses rather than reporting tested effects: trained raters will classify claims with reproducible agreement; claims judged to contain evidence drift will more often lack an explicit bridge than domain-concordant claims; and structured use of the framework will improve the transparency and proportionality of reviews or recommendations compared with usual appraisal. These hypotheses are intended for subsequent empirical testing.

### 2.2. Literature Identification, Eligibility, and Case Selection

Targeted searches were conducted in PubMed/MEDLINE, Scopus, Web of Science, and Google Scholar and updated through 31 July 2026. Searches emphasized ECC literature published from 2010 onward, with backward citation searching for foundational work published before 2010. Search concepts included early childhood caries, vitamin D, Mendelian randomization, dental rehabilitation under general anesthesia, oral microbiome, silver diamine fluoride, sugar-sweetened beverage taxation, food marketing, commercial and structural determinants of health, causal inference, implementation science, knowledge translation, and health equity.

Sources were eligible if they (a) addressed ECC or a foundational methodological framework; (b) included a question, design, outcome, conclusion, or recommendation relevant to one or more domains; and (c) helped identify either a legitimate bridge or a plausible cross-domain overextension. Priority was given to systematic reviews, randomized trials, longitudinal studies, Mendelian-randomization analyses, natural experiments, interrupted time-series and difference-in-differences studies, clinical guidance, and implementation evaluations. Non-ECC sources were retained only for foundational causal, translational, review-method, or validation concepts. Commentary was not used as evidence of intervention effectiveness.

Selection was purposive and contrastive. For each trajectory, the synthesis sought both evidence supporting the signal or intervention and evidence defining its limits—for example, observational and biological evidence alongside Mendelian-randomization or trial findings for vitamin D; SDF efficacy and guidance alongside uncertainty about longitudinal care pathways; and policy evaluations alongside their identifying assumptions, context dependence, and possible substitution effects. Five trajectories were retained because they represented nonredundant transitions and could be analyzed within the same framework. These are illustrative examples, not accusations that every cited study, author, guideline, or policy commits evidence drift.

A detailed tabulation of search strategies for each trajectory is provided in Table 1. Formal record counts for each trajectory were not maintained, as the objective was conceptual case selection rather than systematic enumeration.

To ensure non-redundancy, the following candidate trajectories were considered but excluded from selection: fluoride varnish (where the primary evidence interpretation is less contested in the recent literature, making it less instructive as a contrastive example) and maternal oral health education (where the primary inferential issue is implementation fidelity rather than cross-domain inference). Their exclusion does not imply immunity to drift, only that they offered less conceptual contrast than the five retained cases.

### 2.3. Framework Construction and Analytical Procedure

Framework construction proceeded in four steps: identify the primary question or estimand; assign a primary domain and, where needed, secondary domain tags; compare the domain of the conclusion or recommendation with the domain actually investigated; and specify the bridge and residual uncertainty required for any cross-domain claim. Candidate domain definitions were iteratively compared across the five trajectories and against conceptual-framework, causal-inference, translational-science, and implementation sources [18,20,21,22,23,24,25,26]. No pooled estimates, formal risk-of-bias synthesis, or certainty grading were conducted. The manuscript was structured according to the SANRA reporting domains [17].

## 3. Defining Evidence Drift

Evidence drift is the movement of a finding from one domain of inference to another without adequate methodological or conceptual support. It can occur in individual studies, reviews, guidelines, policy, or public communication. Bridging evidence is the explicit support required for that movement—for example, temporality, a causal identification strategy, intervention evidence, diagnostic assessment, a validated surrogate-to-outcome link, or an implementation evaluation. Inferential fidelity means keeping the conclusion proportionate to the evidence and making any bridge and residual uncertainty visible.

Evidence drift overlaps with, but is not identical to, overinterpretation [27], knowledge translation [22,28], implementation science [23,24], or research waste [29]. Knowledge translation asks how established evidence reaches practice; implementation science asks how an intervention is adopted and sustained. The present framework asks an earlier question: whether the claim being translated is supported by the evidence base that generated it. It is therefore intended to complement, not replace, established appraisal and implementation models.

In ECC research, drift may occur when association is treated as causation, mechanism as effectiveness, consequence as etiology, lesion arrest as disease resolution, or program reach as proof of equity. The final example is a potential implementation inference, not a presumption that access-oriented programs are inequitable. Crossing domains is legitimate when the bridge is explicit and adequate.

ECC is particularly susceptible because a high disease burden, plausible interventions, and pressure for scalable delivery often coexist. The framework does not discourage translation; it makes the required evidence and qualifications explicit.

**Four-Question Evidence Drift Test**

1. Which evidentiary domain does the primary research question address?
  Identify whether the study primarily examines association, mechanism, causation, consequence, disease control, or policy implementation.
2. What domain does the conclusion or recommendation fall within?
  Determine whether the conclusion remains within the domain investigated or advances into another domain.
3. What bridging evidence justifies movement between the domains?
  Look for temporality, causal identification, intervention evidence, diagnostic assessment, implementation evaluation, or another explicit bridge.
4. Are uncertainty, context, and alternative explanations retained?
  Assess whether the conclusion acknowledges relevant assumptions, limitations, effect modification, and the limits of generalizability.

Evidence drift should be suspected when a conclusion crosses evidentiary domains without sufficient bridging evidence or explicit qualification. Crossing domains is not inherently inappropriate; the problem is unsupported crossing.

Evidence drift is a specific cross-domain translation error rather than a synonym for all forms of evidentiary overstatement. It falls within the broader problem of overinterpretation but differs from errors focused on surrogate endpoints, generalizability, implementation, or causal inference. Table 2 summarizes these relationships and clarifies the present framework’s distinct contribution.

## 4. The Causal-Translation Framework

**Figure 1:**
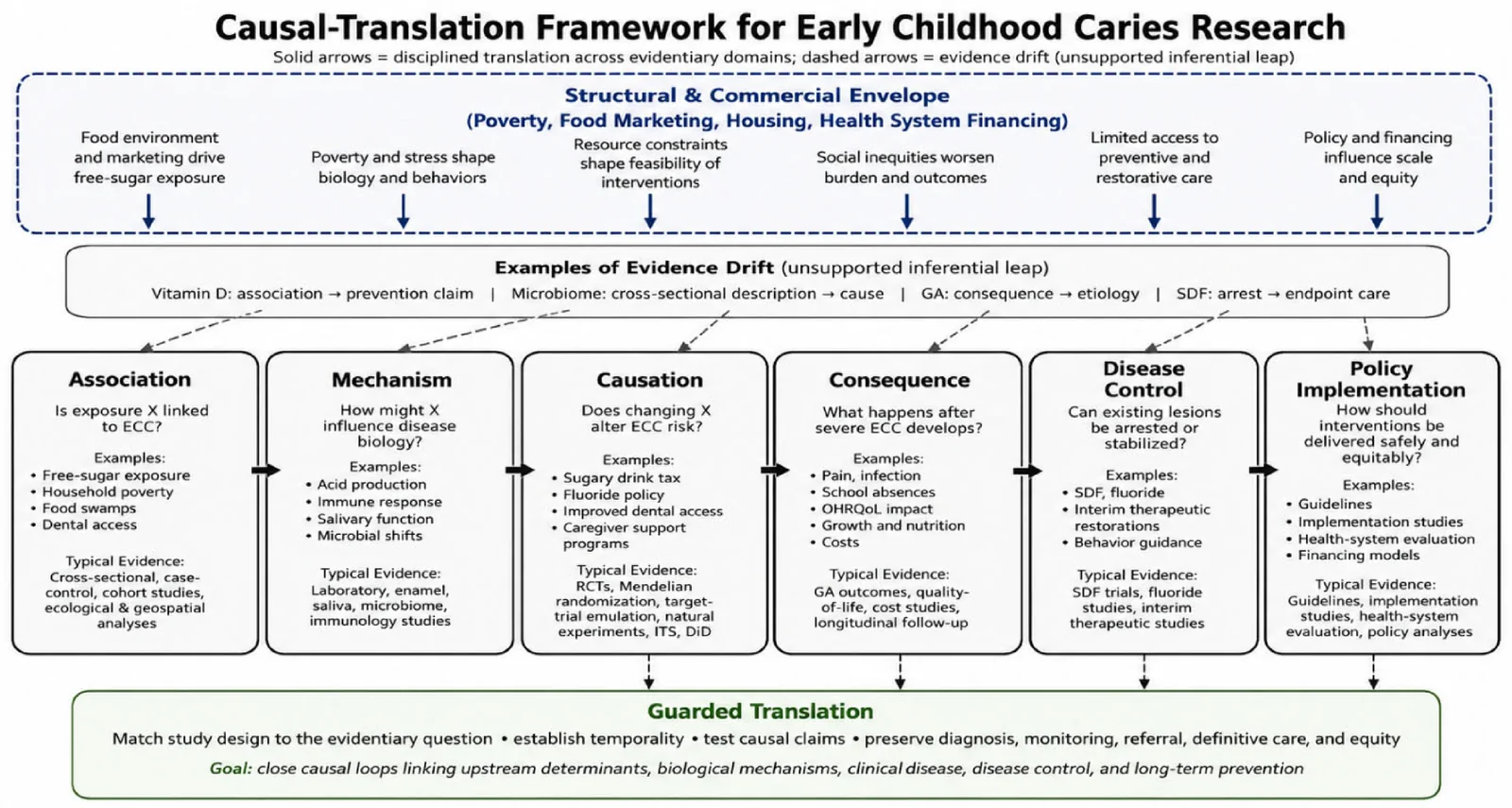
Causal-translation architecture for early childhood caries research. The six domains classify the primary inferential or clinical claim rather than imposing a mandatory linear sequence. Solid arrows indicate transitions supported by appropriate bridging evidence, whereas dashed arrows indicate unsupported inferential leaps. Commercial and structural determinants form a cross-cutting upstream envelope because they generate exposure distributions, modify effects, and shape access and implementation. Feedback loops, multi-domain studies, and reverse translation are expected.

A coherent ECC research program can be organized into six related yet distinct evidentiary domains: association, mechanism, causation, consequence, disease control, and policy implementation. These domains are not a rigid hierarchy, and strong studies may span more than one. Each domain addresses a distinct question and supports a different type of conclusion. Table 3 summarizes the framework, and Figure 1 distinguishes guarded translation from unsupported inferential movement.

The six-domain structure was selected through iterative comparison against three criteria: (a) coverage of the major inferential questions posed in ECC literature, (b) non-overlap of the primary estimand (e.g., mechanism asks ‘how,’ causation asks ‘if changing X’), and (c) alignment with existing translational frameworks while avoiding the conflation of clinical management (consequence/disease control) with population-level delivery (policy implementation). Seven domains were considered but rejected when implementation was split into ‘adoption’ and ‘sustainability’; these were merged into a single policy domain because ECC literature rarely distinguishes them empirically. Five domains were insufficient because they could not separate disease burden documentation (consequence) from active lesion management (disease control), which are routinely confused in SDF and GA studies.

The six-domain structure comprises two linked but non-equivalent triads. The evidence-generation triad—association, mechanism, and causation—addresses whether a signal exists, how it might operate, and whether changing an exposure alters disease risk. The clinical-response and translation triad—consequence, disease control, and policy implementation—addresses the burden of established disease, the management of existing lesions, and the delivery of evidence-informed care within health systems. Commercial and structural determinants cut across both triads by generating exposure distributions, modifying effects, and shaping access and implementation. The framework is therefore functional rather than a hierarchy of evidence levels: its purpose is to make the inferential transition between questions explicit and to identify the bridging evidence required when conclusions cross domains.

The domains classify the primary inferential or clinical question, not the study design itself. No design belongs automatically to only one domain. An SDF randomized trial uses a causal design but may primarily address a disease-control question, whereas a natural experiment evaluating an SSB tax uses causal methods to examine a structural exposure and may also inform policy implementation. Studies may therefore be assigned one primary domain, based on their principal question or estimand, and one or more secondary domain tags. This dual-tagging rule prevents the framework from being mistaken for a mutually exclusive hierarchy of study designs.

The framework synthesizes established causal-inference models [20,21,25,26], translational science [22], and implementation frameworks such as RE-AIM and CFIR [23,24], but adapts them to the claim structure of ECC research. Its novel unit is the inference: the relationship between the question investigated and the conclusion advanced. Unlike caries-risk or treatment taxonomies, it does not rank interventions or designs. It identifies where a claim sits, whether it spans domains, and what bridge would justify that crossing.

Operationally, users assign one primary domain to the principal question or estimand and optional secondary tags to multi-domain studies. They then apply the Four-Question Test to the conclusion or recommendation and document the bridging evidence, assumptions, and remaining uncertainty. This procedure can be used during protocol development, manuscript appraisal, guideline deliberation, education, or policy review; practical applications and proposed outcome measures are detailed in Section 10.

The framework should be used alongside, rather than instead of, design-specific risk-of-bias assessments, certainty-of-evidence methods, and implementation frameworks. A study may be internally rigorous yet still be overextended in translation, while a well-calibrated conclusion may appropriately remain within a limited domain.

## 5. Principles for Preventing Evidence Drift

Preventing evidence drift requires sustained alignment among the research question, evidentiary domain, study design, analytic assumptions, and the strength of the resulting claim. The following principles offer practical safeguards to preserve inferential fidelity across etiological, mechanistic, clinical, disease-control, and policy research.

1. Classify the claim before evaluating the design. The primary domain should be determined by the question and conclusion, not by the study label alone.
2. Associational findings should generate appropriately qualified signals and hypotheses. Decision-relevant causal claims require explicit identification assumptions and appropriate causal evidence.
3. Mechanistic evidence strengthens biological plausibility but does not replace evidence of clinical or population-level effects.
4. Consequence evidence documents disease burden and treatment benefit. It should not be interpreted as explaining disease etiology unless the relevant exposure pathways are measured.
5. Disease-control outcomes should not be equated with etiological prevention, restoration of form and function, or completion of care. Disease-control interventions should remain linked to diagnosis, prevention, monitoring, referral, and definitive care, as appropriate.
6. Policy implementation should be judged by more than reach or uptake. Fidelity, effectiveness, safety, sustainability, referral completion, access to definitive care, and equity should also be assessed.
7. Commercial and structural determinants should be modeled in accordance with the causal question. They may serve as exposures, common causes, mediators, or effect modifiers and should not be treated merely as background context [30,31,32,33,34].

## 6. Taxonomy of Prevention, Stabilization, and Definitive Care

A central way to prevent evidence drift is to use precise language. In this framework, Table 4 provides operational definitions that distinguish prevention, stabilization, lesion arrest, definitive care, and recurrence prevention along the ECC disease trajectory. These definitions are intended to prevent domain confusion rather than to replace formal classification systems.

When applied to cavitated lesions, SDF is primarily a disease-control intervention and, in many pathways, a form of interim stabilization. Lesion arrest is a legitimate disease-control endpoint but does not, by itself, demonstrate etiological prevention, restoration of lost form and function, or completion of care. Dental rehabilitation delivered under general anesthesia (GA) may constitute tertiary prevention and definitive treatment for severe disease; GA itself is an anesthetic modality, not an etiological explanation.

Although developed using ECC examples, the framework may also help interpret emerging evidence across orthodontics, dental traumatology, regenerative dentistry, oral medicine, periodontology, and broader health sciences when observational findings, mechanistic evidence, therapeutic interventions, and policy recommendations coexist.

## 7. Five Illustrative Evidence Trajectories

The cases are intentionally concise. Each identifies a valid contribution, a potential cross-domain claim, and the bridge needed to support that claim. Table 5 provides the comparative detail. These examples are selected to illustrate possible patterns of evidence drift, not to assert that every cited study, author, or recommendation necessarily contains such an error.

### 7.1. Vitamin D: Association Is Not Prevention

Observational studies report associations between vitamin D status and ECC [5,6]. However, the bridge to a prevention claim requires evidence that changing vitamin D status alters ECC risk.

Mendelian-randomization and dose-comparison trial findings do not support broad promotion of supplementation as a stand-alone ECC preventive strategy [7,8,9]. These findings do not address treatment of clinically diagnosed deficiency for general health; they apply only to the ECC-specific prevention claim. ECC-specific recommendations should therefore be confined to populations and developmental windows in which a causal benefit has been demonstrated.

### 7.2. Dental Rehabilitation Under General Anesthesia (Ga): Consequence Is Not an Etiological Explanation

Studies of dental rehabilitation under GA document the burden of severe ECC and show consistent improvements in child and family oral health-related quality of life after comprehensive treatment [35,36]. These findings support timely access to care and quantify consequences; they do not identify why the disease developed or which upstream exposure would have prevented it. Etiological claims require measurement of the relevant behavioral, biological, and structural pathways rather than inference from post-treatment improvement.

### 7.3. Oral Microbiome Research: Description Requires Temporality

Microbiome studies have clarified the ecology of acidogenic and aciduric communities in ECC [10,11,12,13]. Cross-sectional profiles obtained after lesions are established cannot, by themselves, distinguish initiating causes from consequences or mediators of disease. The bridge to causal initiation is temporality and functional evidence: longitudinal sampling before lesion onset, tooth-level analyses, and interventions demonstrating that a specified microbial change alters subsequent disease risk.

### 7.4. Silver Diamine Fluoride: Arrest Is Not Endpoint Care

Empirical evidence supports SDF as a disease-control intervention capable of arresting selected cavitated lesions, though certainty and outcomes vary across populations, protocols, and follow-up [14,15]. That evidence does not, by itself, establish etiological prevention, restoration of lost form and function, comprehensive diagnosis, or completion of care. A separate implementation hypothesis is that endpoint-only SDF pathways could contribute to differential care when used disproportionately among children facing access barriers; direct evidence for that outcome remains limited. Testing the hypothesis requires comparative program evaluations of diagnostic completeness, follow-up, referral, escalation, definitive-care completion, and equity [16,37,38,39].

### 7.5. Sugar Taxation and Food Marketing: Structural Policies Are Not a Substitute for Individual Care

WHO guidance describes fiscal policies and sugar-sweetened beverage taxation as population-level tools whose design, implementation, monitoring, and evaluation require explicit attention [40,41]. Natural experiments and interrupted time-series studies have linked the UK Soft Drinks Industry Levy to product reformulation and lower sugar sales or exposure [42,43], and, in England, to fewer-than-expected childhood hospital admissions for carious tooth extractions [44]. These are important population-level findings, but their interpretation depends on policy design, identification assumptions, substitution, context, and follow-up. The potential drift is not a claim made by the cited studies; it arises if population effects are used to imply that individual prevention, caregiver support, diagnosis, or treatment is no longer needed. Structural policy and clinical care address different parts of the causal system and are complementary.

Table 5 summarizes the contrast among the valid contribution, potential drift, and required correction in each example.

This paper builds upon and extends the SDF-focused analysis previously proposed by the author [37] by generalizing the ‘evidence drift’ concept across five non-redundant ECC trajectories (vitamin D, GA rehabilitation, microbiome, SDF, and sugar taxation) and by introducing a formal six-domain framework with a specific validation agenda.

## 8. Proposed Guardrails and Empirical Questions for SDF Translation

Table 6 separates established SDF evidence from proposed implementation safeguards and measurable indicators. Evidence supports lesion arrest [14,15], while professional guidance addresses case selection, consent, monitoring, and referral [16,38,39]. By contrast, whether specific delivery models create, prevent, or reduce differential standards of care is an empirical question. The guardrails are therefore proposed evaluation criteria, not a validated package, and not evidence that a specific program has caused two-tier care.

These indicators translate a normative concern into a falsifiable implementation question. The concern would be strengthened if access gains coexist with systematically lower diagnostic completeness, follow-up, or definitive-care completion among disadvantaged children; comparable or improved outcomes would weaken it. The framework therefore supports evaluation of SDF pathways without assuming either benefit or harm beyond the measured outcomes.

## 9. Commercial and Structural Determinants as a Cross-Cutting Layer

Commercial and structural determinants are not a seventh sequential domain. Depending on the causal question, poverty, housing, food marketing, product availability, pricing, school food environments, caregiver work conditions, fluoride access, and dental service availability may serve as exposures, common causes, mediators, or effect modifiers [30,31,32,33,34,45]. Their placement must therefore be specified in the causal model. Upstream policies can change exposure distributions, while clinical prevention and treatment remain necessary for children who develop disease. The framework is intended to show how these levels connect without treating one as a substitute for the other.

## 10. Implications and Operational Use

### 10.1. Research Design and Evidence Appraisal

During protocol development, investigators can specify the intended domain, estimand, bridge, and decision-relevant outcome before selecting the design. During evidence appraisal, users can record the research question’s domain, the conclusion’s domain, the evidence supporting any transition, and the uncertainty retained. Proposed audit outcomes include the proportion of claims with domain concordance, the proportion of cross-domain claims with an explicit bridge, and the frequency with which alternative explanations or limits to generalizability are reported.

### 10.2. Peer Review and Guideline Development

Peer reviewers and guideline panels could complete the Four-Question Test independently before discussion, then compare classifications and the reasons for disagreement. The framework addresses domain fit and should accompany, not replace, GRADE certainty assessment and AGREE II guideline-appraisal processes [46,47]. Pilot outcomes could include inter-rater agreement, time to completion, the number of conclusions qualified or recommendations modified, and whether the link between evidence and recommendation becomes more explicit.

### 10.3. Education and Training

In undergraduate, postgraduate, and continuing education, learners can classify paired research questions and conclusions, identify the missing bridge in deliberately overextended examples, and rewrite conclusions to be proportionate to the evidence. Evaluation can measure classification accuracy, agreement with an expert reference standard, confidence, completion time, and transfer to novel ECC scenarios.

### 10.4. Clinical and Policy Implementation

Clinical and policy users can apply the framework to audit what an intervention has demonstrated and what the surrounding pathway must still deliver. For disease-control programs, relevant measures include diagnostic completeness, informed consent, follow-up, referral, pain or disease outcomes, escalation, definitive-care completion, recurrence, caregiver understanding, sustainability, and differential outcomes across groups. The framework does not prescribe a single treatment pathway; it requires that the claim for a pathway match the outcomes actually evaluated.

**Figure 2:**
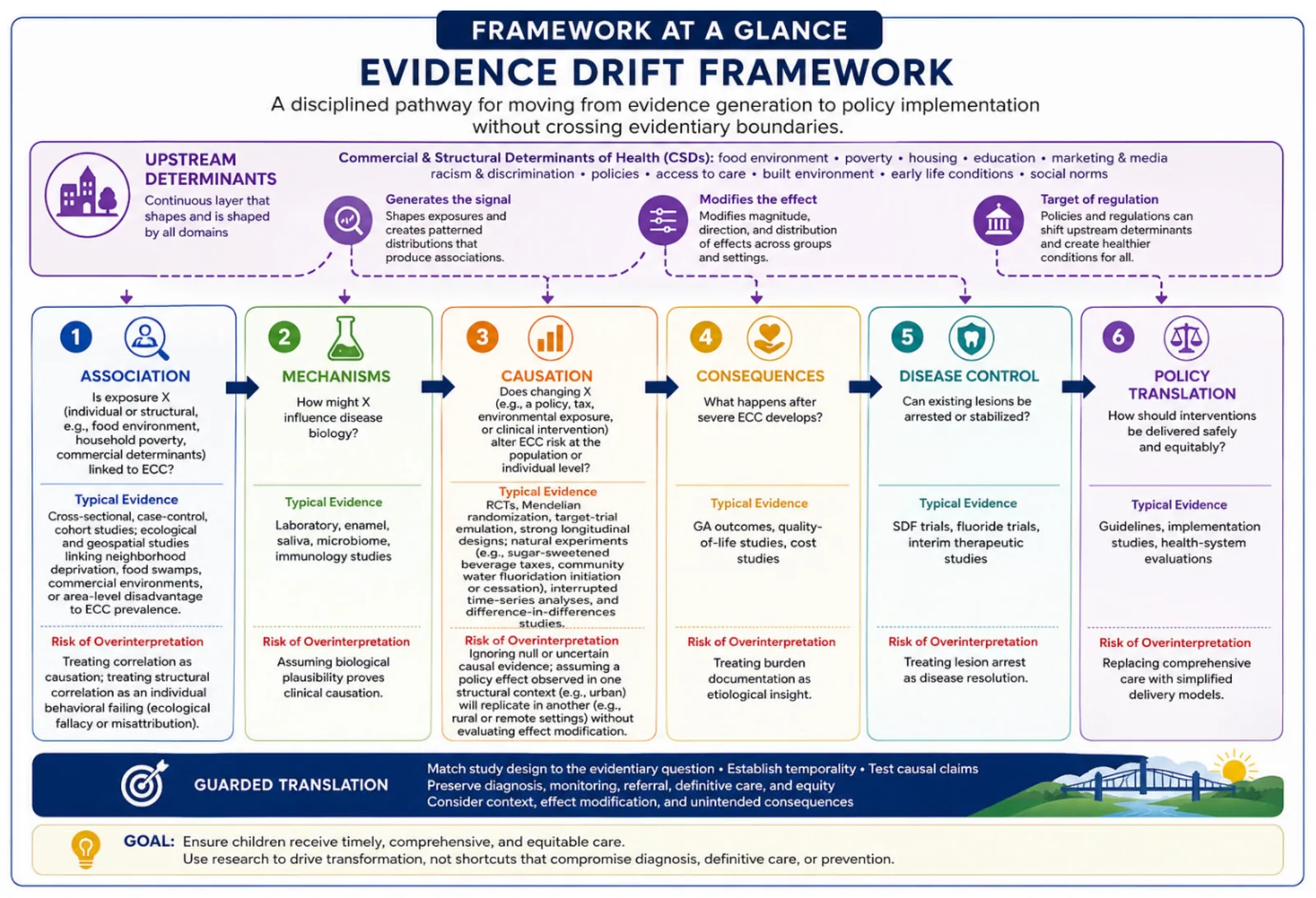
**Framework at a Glance. **This operational summary presents the six evidentiary domains and the Four-Question Evidence Drift Test. The upstream-determinants track is continuous rather than a seventh sequential domain. The figure depicts inferential fidelity as the proportionality between the evidence generated and the conclusion or recommendation advanced.

**Framework at a Glance.** Figure 2 condenses the six domains, the upstream-determinants track, and the Four-Question Test into a one-page reference for research design, appraisal, teaching, guideline deliberation, and policy review.

## 11. Limitations and Empirical Validation

This paper has limitations. It is a single-author conceptual synthesis based on a targeted narrative review, not a systematic review, and source selection was purposive rather than exhaustive. Search yields were not independently screened, coded, or assessed for risk of bias, and no effect estimates were pooled. The five trajectories were selected to maximize conceptual contrast, so they should not be interpreted as a prevalence estimate of evidence drift in ECC.

The framework itself has not been empirically validated. Some claims—especially the possibility that endpoint-only SDF pathways may contribute to differential standards of care—are explicitly presented as prospective implementation hypotheses rather than established outcomes. The examples are weighted toward high-income health systems and may require adaptation in settings where workforce, financing, disease burden, and access differ.

A potential criticism is that the framework may be overly conservative, delaying public health action when evidence is imperfect yet sufficient for decision-making under uncertainty. The framework does not mandate inaction; it mandates transparency. If a policy recommendation is made despite a missing bridge (e.g., implementing a sugar tax based on associative evidence alone), the user must explicitly state the assumptions, uncertainty, and trade-offs. The concern is not translation per se, but unsupported or hidden translation. The framework therefore supports proportionate action when uncertainty is clearly communicated.

Strong studies may span multiple domains, and domain assignment may depend on the stated estimand and the level of inference. The primary/secondary tagging rule is intended to accommodate this complexity, but ambiguity and rater disagreement are expected. The framework should therefore be treated as a transparent heuristic until its definitions, decision rules, reliability, and utility are tested.

Validation can proceed in four phases. First, a two- or three-round Delphi study with pediatric dentists, epidemiologists, causal-inference researchers, implementation scientists, public-health specialists, methodologists, and caregiver representatives can assess content validity and refine definitions using prespecified consensus criteria [48]. Second, blinded raters can classify a purposive sample of ECC studies and recommendations; overall and domain-specific agreement should be reported with confidence intervals using kappa and/or Gwet’s agreement coefficient [49]. Third, construct or known-groups validity can be tested by predicting that examples independently judged to overextend their evidence are more often classified as cross-domain claims lacking a bridge. Fourth, controlled studies in peer review, guideline development, and education can measure completion time, acceptability, interpretive agreement, detection of overextended claims, and changes in recommendation wording or strength [50].

Three prespecified results would support further development: reproducible domain classification, discrimination between calibrated and overextended claims, and improved transparency without a prohibitive time burden. Failure to meet any of these outcomes would require revision or rejection of the relevant domain definitions or decision rules. Only after such testing should the framework be converted into a standardized assessment instrument.

## 12. Conclusions

Evidence drift names a specific translation error: a conclusion moves from the domain under investigation into another domain without an adequate bridge or explicit qualification. The proposed six-domain framework makes that movement visible by separating association, mechanism, causation, consequence, disease control, and policy implementation, while retaining commercial and structural determinants as a cross-cutting causal layer.

This is a conceptual and currently unvalidated contribution. Its immediate value lies in providing a transparent vocabulary and appraisal procedure; its scientific value will depend on testing for empirical reliability, validity, and utility. In ECC research and policy, responsible translation requires authors and decision-makers to state what the evidence demonstrates, what additional bridge supports any broader claim, and what uncertainty remains.

## Figures and Tables

**Table 1:** Search strategies for illustrative evidence trajectories.

Trajectory/Concept	Primary Search Terms (Boolean)	Key Limiters	Supplementary Filters
Vitamin D	(“vitamin D” OR “25-hydroxyvitamin D”) AND (“early childhood caries” OR “ECC” OR “dental caries”) AND (“Mendelian randomization” OR “RCT” OR “trial”)	2010–2026; English	Systematic reviews, MR studies, RCTs
GA Rehabilitation	(“general anesthesia” OR “GA”) AND (“dental rehabilitation” OR “dental treatment”) AND (“early childhood caries” OR “pediatric”) AND (“quality of life” OR “QoL” OR “consequence”)	2010–2026; English	Longitudinal studies, QoL measures
Oral Microbiome	(“microbiome” OR “microbiota” OR “ecology”) AND (“early childhood caries” OR “ECC”) AND (“longitudinal” OR “temporality” OR “tooth-level”)	2010–2026; English	Longitudinal/functional studies
Silver Diamine Fluoride	(“silver diamine fluoride” OR “SDF”) AND (“caries arrest” OR “disease control”) AND (“guidelines” OR “implementation” OR “equity”)	2010–2026; English	Systematic reviews, Clinical guidance
Sugar Taxation	(“sugar-sweetened beverage tax” OR “SSB tax” OR “fiscal policy”) AND (“dental caries” OR “oral health”) AND (“natural experiment” OR “interrupted time series”)	2010–2026; English	Natural experiments, ITS, Diff-in-diff

**Table 2:** Relationship between evidence drift and adjacent interpretive, causal, and implementation concepts *.

Concept	Core Problem	Relationship to Evidence Drift	ECC Illustration	Principal Safeguard
**Evidence drift**	A finding is used to support a stronger or different claim in another evidentiary domain without an adequate bridge or explicit qualification.	The overarching cross-domain translation error addressed by the present framework. It includes transitions beyond causation, such as consequence-to-etiology, disease control-to-completion of care, and implementation reach-to-equity.	SDF lesion arrest is interpreted as etiological prevention, restoration of form and function, completion of care, or proof that a delivery pathway is equitable.	Identify the domain of the research question and the domain of the conclusion; specify the bridging evidence, assumptions, context, and remaining uncertainty.
**Overinterpretation**	Conclusions are stronger, broader, or more certain than the results justify because of spin, selective emphasis, imprecision, or insufficient attention to limitations.	The broader category. Evidence drift is a specific form of overinterpretation in which the overstatement involves movement between evidentiary domains. Overinterpretation can also occur without a domain change.	A small, imprecise, secondary, or statistically nonsignificant effect is described as demonstrating meaningful clinical benefit.	Align the conclusion with the prespecified outcome, effect size, precision, risk of bias, and certainty of evidence.
**Surrogate-endpoint misuse**	An intermediate, biomarker, process, or proxy outcome is treated as if it establishes a patient-important or definitive clinical outcome without a validated relationship.	A specific endpoint-substitution problem that may produce evidence drift when a proxy result is translated into a claim belonging to another domain. It is narrower than evidence drift.	A salivary biomarker, microbial profile, plaque measure, or short-term biological change is treated as proof that future ECC incidence or clinically important disease has been reduced.	Demonstrate that the surrogate reliably predicts the patient-important outcome and, where possible, measure that outcome directly.
**External-validity failure**	Findings are generalized beyond the population, setting, health system, intervention, follow-up period, or delivery conditions actually studied.	Overlaps with evidence drift when context-specific evidence is translated into a broad clinical or policy claim. However, external-validity failure may occur while the claim remains within the same evidentiary domain.	The effect of a sugar tax or SDF pathway in one urban, well-resourced system is assumed to apply to rural, remote, differently financed, or higher-risk populations without examining effect modification.	Define the target population and setting; assess transportability, contextual differences, effect modifiers, implementation conditions, and replication across settings.
**Implementation failure**	An evidence-supported intervention is not adopted, delivered with fidelity, monitored, sustained, or integrated into the surrounding care system.	Primarily a delivery failure rather than an inference error. A valid evidentiary claim can still be followed by implementation failure. Evidence drift occurs when uptake or reach is interpreted as proof of effectiveness, safety, completion of care, or equity.	An SDF program achieves high uptake but has incomplete diagnosis, follow-up, referral, escalation, or definitive-care completion.	Evaluate fidelity, reach, effectiveness, safety, follow-up, referral completion, sustainability, acceptability, and differential outcomes across population groups.
**Causal overclaiming**	Associational or mechanistic evidence is described as proving that an exposure causes, prevents, or changes disease without an adequate causal identification strategy.	A common and important subtype of evidence drift, usually involving movement from association or mechanism into causation. Evidence drift is broader because it also includes noncausal clinical and policy transitions.	Vitamin D associations are used to recommend supplementation for ECC prevention, or cross-sectional microbiome profiles are interpreted as causes of disease initiation.	Establish temporality; address confounding, selection, and reverse causation; use appropriate causal designs, natural experiments, randomized trials, or triangulation of evidence.

* Note: These concepts are related but not mutually exclusive. A single claim may simultaneously involve, for example, causal overclaiming, surrogate-endpoint misuse, and external-validity failure. Evidence drift identifies the overarching mismatch between the domain supported by the evidence and the domain occupied by the conclusion or recommendation.

**Table 3:** Six-domain causal-translation framework for ECC research.

Domain	Core Question	Typical Evidence	Risk of Overinterpretation
**Association**	Is exposure X (individual or structural, e.g., food environment, household poverty, commercial determinants) linked to ECC?	Cross-sectional, case–control, cohort studies; ecological and geospatial studies linking neighborhood deprivation, food swamps, commercial environments, or area-level disadvantage to ECC prevalence.	Treating correlation as causation; treating structural correlation as an individual behavioral failing (ecological fallacy or misattribution).
**Mechanism**	How might X influence disease biology?	Laboratory, enamel, saliva, microbiome, immunology studies	Assuming biological plausibility proves clinical causation
**Causation**	Does changing X (e.g., a policy, tax, environmental exposure, or clinical intervention) alter ECC risk at the population or individual level?	RCTs, Mendelian randomization, target-trial emulation, strong longitudinal designs; natural experiments (e.g., sugar-sweetened beverage taxes, community water fluoridation initiation or cessation), interrupted time-series analyses, and difference-in-differences studies.	Ignoring null or uncertain causal evidence; assuming a policy effect observed in one structural context (e.g., urban) will replicate in another (e.g., rural or remote settings) without evaluating effect modification.
**Consequence**	What happens after severe ECC develops?	GA outcomes, quality-of-life studies, cost studies	Treating burden documentation as etiological insight
**Disease Control**	Can existing lesions be arrested or stabilized?	SDF trials, fluoride trials, interim therapeutic studies	Treating lesion arrest as disease resolution
**Policy Implementation**	How should evidence-informed interventions be delivered, monitored, adapted, and sustained safely and equitably	Guidelines, implementation studies, process evaluations, health-system evaluations, equity audits	Equating reach or uptake with effectiveness or equity; replacing comprehensive care with simplified pathways

**Table 4:** Taxonomy of prevention, stabilization, and definitive care in ECC.

Term	Definition in ECC Care
**Primary prevention**	Preventing disease onset through fluoride exposure, dietary interventions, oral hygiene support, community water fluoridation, and policies that reduce sugar exposure.
**Secondary prevention**	Early detection and early intervention before the disease progresses.
**Tertiary prevention**	Reducing the impact of established disease, including restorative care, rehabilitation, recurrence prevention, and complication management.
**Interim stabilization**	Temporary disease management intended to slow progression until definitive care is available.
**Caries arrest**	Halting progression of an existing cavitated lesion.
**Definitive restorative care**	Treatment that restores form, function, and, where possible, esthetics, such as restorations, crowns, pulp therapy, or extraction when indicated.
**Recurrence prevention**	Preventing new lesions or recurrent disease after stabilization or definitive care.

**Table 5:** Evidence drift across five ECC case examples.

Case	Valid Contribution	Potential Drift	Framework Correction
**Vitamin D**	Identifies observational associations between vitamin D status and ECC risk.	Vitamin D supplementation is promoted as a stand-alone ECC prevention strategy based primarily on observational associations.	Require stronger causal evidence (e.g., Mendelian randomization, randomized trials, and subgroup-specific analyses) before making preventive recommendations.
**Dental rehabilitation under general anesthesia (GA)**	Documents disease burden and demonstrates improvements in oral health-related quality of life following comprehensive treatment.	Consequence research is interpreted as explaining the etiology of ECC rather than documenting its burden.	Use GA outcome studies to advocate for timely care while linking findings backward to upstream risk pathways and forward to recurrence prevention and long-term disease management.
**Oral microbiome**	Describes microbial dysbiosis and provides biologically plausible mechanisms underlying ECC.	Cross-sectional microbial profiles are interpreted as establishing causal mechanisms for disease initiation.	Prioritize longitudinal, functional, and tooth-level studies that establish temporality and distinguish causes from consequences of disease.
**Silver diamine fluoride (SDF)**	Shows that SDF can arrest selected existing cavitated lesions and stabilize disease in appropriate cases.	Lesion arrest is interpreted as etiological prevention, endpoint care, or proof that an endpoint-only delivery model is equitable.	Separate established lesion-arrest evidence from prospective implementation risks; evaluate diagnosis, monitoring, referral, escalation, definitive-care completion, and equity.
**Sugar taxation and food marketing (Commercial and Structural Determinants of Health)**	Natural experiments and quasi-experimental studies associate taxation with product reformulation, lower sugar exposure, and selected population oral-health outcomes.	Population-level effects are interpreted as making individual prevention, caregiver support, diagnosis, or clinical care unnecessary.	Treat structural policy and clinical care as complementary; retain identifying assumptions and evaluate implementation, substitution, equity, sustainability, and reinvestment.

Note: Evidence sources for each case are cited in Section 7.1, Section 7.2, Section 7.3, Section 7.4 and Section 7.5.

**Table 6:** Proposed SDF pathway components and illustrative evaluation indicators.

Category	Proposed Pathway Components	Illustrative Evaluation Indicators
**Clinical**	Tooth-level assessment; assessment for pulpal pathology; treated teeth and surfaces documented; informed consent covering staining, alternatives, and intended role.	Proportion with complete diagnostic documentation; consent quality; inappropriate-treatment or adverse-event rate.
**System**	Prespecified follow-up; referral and care coordination; escalation criteria; pathway to definitive care when indicated.	Follow-up attendance; referral completion; time to escalation; definitive-care completion.
**Equity**	Same diagnostic and eligibility standards across groups; accessible communication; stratified monitoring.	Differences by socioeconomic status, geography, race/ethnicity, disability, or payer in diagnosis, follow-up, referral, and definitive care.
**Quality assurance**	Training and competency assessment; supervision; record audit; adverse-event monitoring; program review.	Competency completion; audit adherence; adverse events; protocol deviations; corrective actions.

## Data Availability

No new data were created or analyzed. The search strategies for the illustrative trajectories are provided in Table 1 of the main text.
